# Detection of Antibiotic Residues in Milk and Public Awareness Level Assessment in Selected Districts of the Sidama Region, Ethiopia

**DOI:** 10.1155/vmi/1579502

**Published:** 2026-05-12

**Authors:** Mishamo Sulayeman, Naod Teferi, Belachew Bacha, Merga Daba

**Affiliations:** ^1^ Faculty of Veterinary Medicine, Hawassa University, P.O. Box 05, Hawassa, Ethiopia, hu.edu.et; ^2^ Department of Animal Health, Alage Agricultural TVT College, Alage, Oromia, Ethiopia; ^3^ Animal Product and Input Quality Testing Center, Ethiopian Agriculture Authority (EAA), Addis Ababa, Ethiopia; ^4^ College of Pastoral Agriculture Science and Technology, Lanzhou University, Lanzhou, 730020, China, lzu.edu.cn

**Keywords:** antibiotic residues, dairy cow, Ethiopia, milk, Sidama

## Abstract

**Introduction:**

Antibiotic residues in milk are a growing public health concern, primarily due to their role in fostering antimicrobial resistance. Consuming contaminated milk can pose significant health risks, especially in areas with weak regulatory systems and limited public awareness.

**Materials and Methods:**

A cross‐sectional study was conducted from September 2023 to June 2024 to assess antibiotic residues in milk and stakeholder awareness. A total of 324 raw milk samples were randomly collected from dairy farms in Hawassa City (198 samples) and Wondo Genet woreda (126 samples). Samples were initially screened using the Delvotest SP sensor kit, and positives were confirmed by high‐performance liquid chromatography (HPLC). Additionally, structured questionnaires were administered to 60 dairy farmers to evaluate their knowledge and awareness of antibiotic residue.

**Results:**

Out of 324 milk samples tested, 29 (9%) were positive for antibiotic residues using the Delvotest SP kit. (HPLC confirmed oxytetracycline (OTC) in 8 (2.5%) samples and penicillin G (PnG) in 16 (4.9%) samples. The mean OTC concentration was below the Codex Alimentarius maximum residue limit (MRL), although some samples exceeded the limit (up to 122.76 µg/L), while PnG residues (up to 142.38 µg/L) markedly exceeded the MRL, indicating potential public health risks and regulatory noncompliance. Previous treatment history, adherence to the withdrawal period, and owner awareness of drug residues were significantly associated with the prevalence of drug residues. Samples from cows with recent antibiotic treatment showed significantly higher residue levels. Among 60 dairy farmers surveyed, only 8.3% were aware of antibiotic residues in milk. All veterinarians (100%) used broad‐spectrum antibiotics for undifferentiated cases, mostly relying on clinical judgment rather than laboratory diagnosis, increasing the risk of drug residues and antimicrobial resistance.

**Conclusion:**

The presence of antibiotic residues in milk and low awareness among dairy farmers in the study areas highlight a critical public health gap. Farmers’ awareness of antibiotic residues was mainly influenced by education level, while veterinarians showed good knowledge of antibiotic risks, but gaps remain in laboratory diagnosis, record keeping, and regulatory understanding, indicating the need for targeted training and stronger residue control.

## 1. Introduction

Milk is a vital component of the human diet, consumed by individuals of all ages due to its high bioavailability and essential contributions to daily nutrient intake [1]. For over five decades, antibiotics have been widely utilized in dairy cattle production for the treatment and prevention of diseases, as well as to enhance milk production and improve feed efficiency [2]. Following their introduction in the late 1940s, antimicrobials became a common treatment for infections in veterinary medicine [3]. However, the extensive and often unregulated use of veterinary antibiotics has led to the presence of antibiotic residues in animal‐derived food products such as milk, meat, and eggs [4].

Antimicrobials, whether synthetic or natural, inhibit or destroy microorganisms, while antibiotics specifically target bacteria [5]. Their widespread use in food‐producing animals has accelerated the emergence of antimicrobial resistance, a major global health threat [5]. Consumption of milk containing antibiotic residues may lead to allergic reactions, cancer, kidney and liver damage, reproductive and immune disorders, and bone marrow toxicity [6, 7]. These residues also interfere with fermentation, disrupting the production of yogurt and cheese and causing economic losses [7].

Antibiotic residues in animal‐derived food are a growing global concern [8]. Antibiotics, classified as bacteriostatic or bactericidal, with narrow or broad spectra, have been used in animals shortly after their adoption in human medicine [9]. Today, around 80% of food‐producing animals receive antibiotics during their lifetime [10]. This widespread use has driven productivity but created heavy reliance on antimicrobials, especially antibiotics [11].

Antibiotics in livestock are used therapeutically, prophylactically, and as growth promoters. Indiscriminate use leads to residues in animal products, posing health risks. In Ethiopia (2015–2017), oxytetracycline (OTC) and penicillin G (PnG) dominated veterinary use, with over 112 million and 43 million defined daily doses, respectively; this is due to low cost and availability. Tetracycline residues can impair liver, kidney, and gastrointestinal functions [12]. Antibiotic resistance results in treatment failures, severe disease, longer hospital stays, higher costs, and increased mortality [13].

In Ethiopia, the use of antimicrobials in livestock is guided by national veterinary drug policies overseen by the Ethiopian Food and Drug Authority. The authority is responsible for registering and licensing veterinary drugs and regulating their distribution, with most antimicrobials intended to be used under veterinary prescription. These regulations also encourage good livestock management practices, such as responsible antimicrobial use and adherence to drug withdrawal periods, to protect public health. However, gaps in implementation remain, particularly within informal and smallholder production systems.

Ethiopia produces an estimated 4.5–4.7 million tons of milk annually, with the Sidama Region contributing about 6%–8% of this total. Within the region, Hawassa City and Wondo Genet districts stand out as important periurban dairy hubs, supplying milk and dairy products to a large share of the local population. Together, these areas contribute roughly 1%–2.5% of the national milk output (approximately 55,000–110,000 tons per year), highlighting their growing role in supporting urban and surrounding communities [14].

Limited studies have detected drug residues, such as OTC and PnG, in cow’s milk [15]. Despite this importance, no prior research has specifically examined antibiotic residues in milk from these districts, leaving a critical gap in understanding their prevalence, sources, and potential impacts on public health and the environment. Addressing this gap is essential for ensuring food safety and protecting community health. Therefore, this study aimed to determine the prevalence of antibiotic residues in milk from the Sidama Region and to identify associated risk factors in milk samples intended for human consumption. Additionally, it seeks to quantify antibiotic concentrations in positive samples and assess the awareness of dairy farmers and veterinary professionals regarding antibiotic residues in milk. The findings will provide vital data to enhance food safety and inform public health initiatives.

## 2. Materials and Methods

### 2.1. Description of Study Area

**FIGURE 1:**
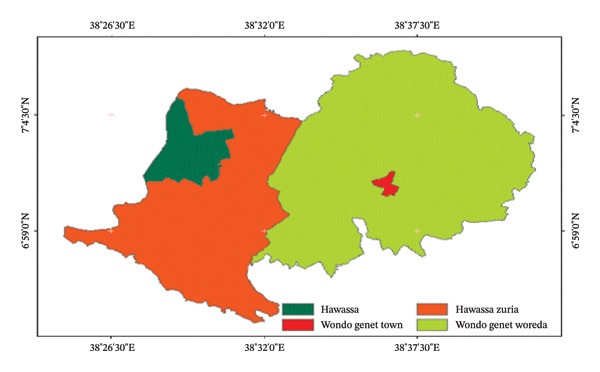
Map of study areas, developed from Ethiopian shape files using QGIS software.

The study was conducted in Hawassa City and Wondo Genet. Hawassa is the capital city of the Sidama region, located 275 km south of Addis Ababa. Geographically, it is located between latitudes 4° 27′ and 8° 30′ north and longitudes 34° 21′ and 39° 1′ east (Figure 1). Wondo Genet is 1723 m above the sea level and is situated in the Sidama region of Southern Ethiopia. Its latitude and longitude are 7°1′N and 38°35′E.

### 2.2. Study Design

A cross‐sectional study was conducted from September 2023 to June 2024. The study focused on bulk milk tanks located in dairy farms in Hawassa City and the Wondo Genet districts.

### 2.3. Sampling Method and Sample Size Determination

A stratified random sampling method was employed to collect milk samples from communal tanks, where milk from all cows was pooled and stored. An estimated prevalence of 12% was used to determine the initial sample size of 162, calculated using the formula provided by Thrusfield [14]. To enhance precision, the sample size was doubled (324) and proportionally distributed between the two districts, with 198 samples from Hawassa City and 126 from Wondo Genet. These milk samples were randomly collected from dairy farms across both districts.

### 2.4. Data Collection

#### 2.4.1. Milk Sample Collection

Approximately 20‐mL bulk tank milk samples were collected from dairy farms using conical centrifuge tubes. Each sample was labeled and identified with the sampling date and a unique identification code. All milk samples were transported to the Hawassa University Veterinary Public Health and Microbiology laboratory in an icebox for qualitative screening using the Delvotest SP. Delvotest‐positive samples were subsequently sent to Animal Product and Input Quality Testing Center, Addis Ababa, which is quality control laboratory of Ethiopian Agricultural Authority (EAA) using high‐performance liquid chromatography (HPLC).

#### 2.4.2. Questionnaire Survey

The awareness and knowledge of respondents were assessed through a questionnaire survey conducted among 60 randomly selected farmers, given the similarity of production systems in the study areas. The questionnaire, which included both closed‐ and open‐ended questions, was also administered to 20 randomly selected participants comprising veterinary professionals working in the study areas and farmers. It covered key aspects such as awareness of antibiotic use, farm management practices, withdrawal periods, and antimicrobial residues, as well as the qualifications of those administering drugs, types and frequency of medication use, record‐keeping practices, and other potential risk factors for contamination.

##### 2.4.2.1. Detection of Antibiotic Residue in Milk Using Delvotest SP Assay

Screening tests were conducted using a commercial Delvotest SP kit (DSM, China). The test is made of an agar gel containing a standard number of bacterial spores (*Bacillus stearothermophilus*) and a pH indicator bromocresol purple. Briefly, 100 µL of milk sample was transferred to the ampoule containing nutrient agar embedded with *Bacillus stearothermophilus* spores and bromocresol purple indicator and incubated at 64°C for 3 h. A clear color change from purple to yellow indicates that the antimicrobial compounds are below the detection limits (negative result). A purple color indicates the presence of antibiotics at or above the detection limits of the test (positive result).

### 2.5. Confirmatory Detection Using HPLC

#### 2.5.1. Sample Extraction and Clean‐Up

The frozen samples (−20°C) were allowed to thaw overnight at 4°C for analysis. Subsequently, 7 mL of the homogenized raw cow milk was pipetted into a 50 mL falcon tube for extraction. Quality control samples of raw cow milk, previously verified to be free from target antibiotics, were spiked with appropriate volumes of a working standards solution for each calibration level, as detailed in Table 1 and annex 3. These spiked samples were prepared in triplicates to facilitate the calibration curve and precision preparation.

**TABLE 1:** Levels of antimicrobial residues determined in cow milk samples in the study areas.

Concentration of residues	Hawassa and Wondo Genet districts (*n* = 324)			
	Sulfonamides	Tetracyclines		Beta lactams
	SDZ	OTC	TTC	PnG
Min. (µg/L)	ND	2.91	ND	1.02
Max. (µg/L)	ND	122.76	ND	142.38
Mean (µg/L)	ND	42.67	ND	26.53
Range (µg/L)	ND	2.91–122.76	ND	1.02–142.38
Total no. (%)	—	8 (2.5%)	—	16 (4.9%)

The extraction and clean‐up of homogenized raw milk were performed with minor adjustments to the procedure described by [16]. The required volume of the working standard solution for each calibration level was calculated and spiked based on the reference standard concentration, followed by the addition of 7 of 0.1‐mL Na2EDTA/McIlvaine buffer to each level. Each calibration level and sample was vortexed for 30 s, after which 2 mL of 0.1% formic acid in acetonitrile was added and shaken for 5 min.

The samples then centrifuged at 4500 rpm for 15 min at +4°C. The upper layer of the butter formed during centrifugation removed carefully with individual spatulas. The supernatant was loaded onto previously activated Oasis HLB 6 cc (200 mg) extraction cartridges, which had been activated with 3 mL of methanol and 2 mL of deionized water. Antibiotic residues eluted from HLB cartridges with 3 mL of methanol, and the elution solvent was evaporated to near dryness under a nitrogen concentrator stream at 35°C and 12 psi. The dried residue was reconstituted with 250 μL of a water:acetonitrile mixture (80%:20%), centrifuged again at 4500 rpm for 5 min at +4°C, and then the final extract was transferred into an HPLC glass vial. Finally, 20 μL of the extract was injected into the HPLC system for chromatographic separation and measurement.

### 2.6. Data Management and Analysis

Data were coded in Excel and analyzed using SPSS v27. Descriptive statistics were presented as percentages. Chi‐square tests assessed associations between risk factors (e.g., awareness, treatment history, record‐keeping, and withdrawal violations) and antibiotic residues, with significance set at *p* < 0.05 and 95% confidence intervals. The method has been shown to be reliable, accurate, and robust for the quantitative determination of targeted drug residues in milk samples [17].

## 3. Results

### 3.1. Qualitative Analysis of the Delvotest SP Assay

The prevalence of antibiotic residues in milk samples, determined using qualitative methods, was 9% (29/324). Notably, a higher prevalence was recorded in Hawassa City, with 10.1% (20/198) of samples testing positive, compared to 7.14% (9/126) in Wondo Genet Woreda, while the difference was not statistically significant (*p* > 0.5).

### 3.2. Quantitative Analysis by HPLC

Out of 29 milk samples that tested positive on Delvotest, quantitative analysis using HPLC identified 24 (8 samples with OTC and 16 samples with PnG were confirmed to have antibiotic residues) with different concentration levels (Table 1). Comparing these results with the standards, the mean OTC level was below the Codex Alimentarius maximum residue limit (MRL), though the maximum concentration (122.76 µg/L) exceeded the limit in a few samples. In contrast, the detected PnG residues (142.38 µg/L) substantially exceeded the MRL, indicating potential public health concerns and regulatory noncompliance.

#### 3.2.1. Risk Factors

Prior treatment with OTC or penicillin was strongly associated with positive drug residue results (100% positive rate) compared to cows that did not receive any treatment (1% positive rate, *p* ≤ 0.001). Owner awareness of drug residues was also significantly associated with positive results (*p* ≤ 0.001). Cows owned by individuals unaware of drug residues had a higher prevalence of positive results (23%) compared with those owned by individuals who were aware (2%) (Table 2).

**TABLE 2:** The effect of contributing factors for the prevalence of antibiotic residues on milk in Wondo Genet and Hawassa City dairy farms.

Variables	Categories	Positive	Prevalence (%)	*p* value	(Chi)^2^
Previous treatment history	OTC	8	33.3	0.001	287.5584
	Pen‐Strep	16	66.7		

Keeping withdrawal period	No	20	83	0.001	22.3099
	Yes	4	16.7		

Record keeping	No	15	62.5	0.487	0.4835
	Yes	9	37.5		

Dairy management	Good	5	20.8	0.335	2.1887
	Intermediate	15	62.5		
	Poor	4	16.7		

Owner awareness on drug residue	No	22	91.7	0.001	36.8636
	Yes	2	8.3		

### 3.3. Result for Questionnaire Survey

#### 3.3.1. Awareness Levels Regarding Drug Residues

Only 8.3% of the respondents have awareness about drug residue, which favors the existence of drug residues in milk intended for human consumptions. The primary antibiotics used on these farms include Pen‐Strep (50%) and OTC (38.3%), suggesting a reliance on specific treatments (Table 3).

**TABLE 3:** Awareness of antibiotic usage in dairy farmer respondents.

Variables	Category	Frequency (%)
Awareness about antibiotic residue in milk	Yes No	5 (8.3) 55 (91.7)

What type of antibiotics do you use in your dairy farm?	Pen‐Strep Oxytetracycline Sulfa drugs	30 (50) 22 (38.3) 8 (11.7)

Commonly used route of drug administration	Intramuscular Intramammary Intrauterine	39 (65) 18 (30) 3 (5)

Do you use treatment record keeping?	Yes No	6 (10) 54 (90)

Do you use separate equipment for milking of treated cows	Yes No	21 (35) 39 (65)

Do you use antibiotic test kit?	Yes No	0 (0) 60 (100)

Who should be responsible for ensuring that food product is free from antibiotic residues	Government regulatory agencies Government regulatory agencies and producers Producers.	23 36 1

Most commonly encountered diseases in your dairy farm?	Mastitis Metritis and LSD Other	32 24 4

Which disease poses the greatest economic impact on your dairy?	Mastitis Metritis	55 5

What type of person administers the treatment for cows?	Veterinarian Assistant veterinarians Dairy farmers	3 23 7

Do you use dry cow intramammary antibiotic treatment?	Yes No	17 43

Participation in any training about dairy farm management	Yes No	15 45

Do you keep to withdrawal periods?	Yes No	20 40

The assessment of 20 veterinarians showed that all respondents (100%) commonly used broad‐spectrum antibiotics for undifferentiated cases, a practice that may increase the risk of drug residues and antimicrobial resistance. While almost all veterinarians calculated drug doses based on body weight, only a few relied on laboratory diagnostics, indicating that treatment is often based on clinical judgment rather than confirmatory testing, which may contribute to residue‐related risks (Table 4).

**TABLE 4:** Awareness of veterinarians on drug residues and rational drug use.

Awareness area of the veterinarians	Response	Frequency (%)
Awareness of risks of using broad‐spectrum antibiotics without clear diagnosis	Yes	20 (100)
	No	0 (0)

Awareness of antimicrobial resistance due to misuse of antibiotics	Yes	18 (90)
	No	2 (10)

Awareness of importance of withdrawal period	Yes	18 (90)
	No	2 (10)

Awareness of MRLs	Yes	12 (60)
	No	8 (40)

Awareness of public health risks of drug residues in animal products	Yes	17 (85)
	No	3 (15)

Awareness of correct dose calculation based on body weight	Yes	19 (95)
	No	1 (5)

Awareness of importance of proper drug use record keeping	Yes	13 (65)
	No	7 (35)

Awareness of national regulations on veterinary drug use	Yes	11 (55)
	No	9 (45)

Awareness of informing farmers on withdrawal period	Yes	15 (75)
	No	5 (25)

Awareness of laboratory diagnosis before treatment	Yes	12 (60)
	No	8 (40)

## 4. Discussion

The current overall prevalence of 7.4% (24/324) for antibiotic residues in the dairy cow milk samples was comparable with previous studies [18, 19] in Kenya and Nepal with indicated prevalence rates of 9% and 9.9%, respectively. Relatively higher prevalence of antibiotic residues, with 12%, was detected in dairy farms located in Tiyo, Digelu Tijo [20], and Nazareth [21]. Similarly, studies conducted in Iran [22, 23] documented even higher contamination rates, reporting prevalence levels of 21% and 23%, respectively. In contrast, lower prevalence rates were reported in Ankara, Turkey (1.3%) [24] and in Brazil, it was reported that (4.3%) [25] milk samples had a detectable level of residue for antibiotic. This inconsistence can be attributed to differences in national legislation regarding antimicrobial use, laboratory testing methodologies, farm management practices, sample sizes, and cultural factors [24].

Out of the 29 milk samples that tested positive by Delvotest, HPLC confirmed the presence of antibiotic residues in 24 samples, specifically 8 with OTC and 16 with PnG. These results reflect the common use of these antibiotics in dairy farms and align with earlier findings in Ethiopia and Iran [20, 21]. The detection of OTC and PnG residues suggests that these antibiotics are commonly used in the study areas, potentially reflecting improper withdrawal practices or a lack of awareness among farmers. Their presence in milk poses significant public health risks, including allergic reactions, antibiotic resistance, and disruption of dairy fermentation processes, emphasizing the need for routine monitoring and farmer education.

The presence of antimicrobial residues in milk from the study areas indicates a continued risk of drug contamination in dairy products. When compared with the MRL set by the Codex Alimentarius Commission, the average level of OTC was within the acceptable limit of 100 µg/L. However, the highest recorded value (122.76 µg/L) exceeded this limit in some samples. In contrast, PnG was detected at levels as high as 142.38 µg/L, which is far above the recommended limit of 4 µg/L. This is concerning, as even small amounts of beta‐lactam residues can cause allergic reactions in sensitive individuals and may contribute to antimicrobial resistance.

In this study, previous treatment history, observance of the withdrawal period, and farmers’ awareness of drug residues showed a clear link with the presence of antimicrobial residues in milk. The 100% positivity observed among previously treated cows appears unusually high and may be influenced by the small number of animals in this subgroup or potential sampling bias. Therefore, this finding should be interpreted with caution. Similar patterns have been reported elsewhere, where skipping or shortening withdrawal times after treatment was a key reason for contamination [26, 27]. Farmers who treat animals without proper guidance often risk leaving residues due to incorrect dosing or early milking [28]. Limited awareness about the health impacts such as allergic reactions and the spread of antimicrobial resistance further compounds the problem [29]. These findings highlight the need for practical training and stricter follow‐up to promote safe drug use and protect consumers.

The questionnaire survey conducted during the study period included questions that were helpful to gain insights into farm management practices and knowledge of dairy farm owners associated with antibiotic usage and antibiotic residues in Sidama, Ethiopia. Knowledge of antibiotic use is a crucial step in the prevention of antibiotic residue presence in animal byproducts. Despite huge efforts of the government and nongovernment institutions to promote and improve livestock management practices in the areas, this study highlighted that the general knowledge of antibiotic residues among the dairy farm owners was somewhat encouraging; however, farm management practices were not in line with their knowledge. Similar results were reported by Abebew et al. (2014) in a questionnaire survey in Debre Zeit and Nazareth.

Farmers’ awareness of antibiotic residues in the study areas was mainly shaped by the education level, with higher education associated with better awareness, while other demographic factors had limited influence [30]. Similarly, the assessment of 20 veterinarians showed good knowledge of antibiotic risks; however, gaps remained in laboratory diagnosis, record keeping, and regulatory understanding. These gaps may be linked to limited laboratory facilities, shortage of trained personnel, and weak regulatory enforcement, consistent with previous studies [31].

The findings highlight the importance of education in raising awareness about antibiotic residues among dairy farmers. With a significant proportion of respondents lacking formal education, there is a clear need for targeted educational initiatives aimed at improving knowledge about safe antibiotic practices and their implications for milk safety. This study underscores the necessity of integrating educational programs into veterinary and agricultural extension services to inform farmers about the risks associated with antibiotic use, promoting safer practices in dairy farming. Improving awareness in this area is crucial for enhancing public health and food safety by reducing the likelihood of antibiotic residues in milk.

## 5. Conclusion and Recommendations

In the study areas, 7.4% of milk intended for human consumption was found to contain antibiotic residues, which raises an important public health concern, especially since PnG exceeded the MRL and OTC was also detected at lower levels. These residues are likely associated with gaps in stakeholder awareness, including limited awareness of the risks of using broad‐spectrum antibiotics without clear diagnosis and the use of multiple antibiotics, all of which may result in milk being consumed before the recommended withdrawal periods are completed. Farmers’ awareness of antibiotic residues was mainly influenced by their level of education, while other demographic factors had little effect. Although veterinarians showed good awareness of antibiotic risks and correct dosing, gaps remain in the importance of laboratory diagnosis before treatment, record keeping, and regulatory understanding, highlighting the need for focused training and stronger residue control efforts. However, these conclusions should be interpreted cautiously due to limited information on how the Ethiopian milk value chain is structured and operates. Strengthening regulatory enforcement, improving stakeholder awareness particularly in laboratory diagnosis, drug withdrawal periods, record keeping, and regulatory compliance, and enhancing monitoring systems are essential to ensure milk safety and protect public health.

**Nomenclature**
BB: Balachew Bacha
MB: Merga Daba
MS: Mishamo Sulayeman
ND: Naod Teferi

## Author Contributions

Mishamo Sulayeman: contributed to conception of the research idea, designing and data collection, data analysis, interpretation of data, and writing and editing of the manuscript. Naod Teferi: contributed to the study concept, interpretation of data, and editing or reviewing of the manuscript. Belachew Bacha: contributed to conception of the research idea, data analysis, and interpretation of data. Merga Daba and Belachew Bacha: contributed to conception of the research idea.

## Funding

The cost of this research work was covered by Hawassa University.

## Disclosure

All authors have read and approved the final manuscript.

## Consent

The authors have nothing to report.

## Conflicts of Interest

The authors declare no conflicts of interest.

## Data Availability

The datasets used and/or analyzed during the current study available from the corresponding author on reasonable request.
